# Single-cell individualized electroporation with real-time impedance monitoring using a microelectrode array chip

**DOI:** 10.1038/s41378-020-00196-0

**Published:** 2020-10-19

**Authors:** Zhizhong Zhang, Tianyang Zheng, Rong Zhu

**Affiliations:** State Key Laboratory of Precision Measurement Technology and Instruments, Department of Precision Instrument, Tsinghua University, Beijing, 100084 China

**Keywords:** Electrical and electronic engineering, Chemistry

## Abstract

The ability to precisely deliver molecules into single cells while maintaining good cell viability is of great importance to applications in therapeutics, diagnostics, and drug delivery as it is an advancement toward the promise of personalized medicine. This paper reports a single-cell individualized electroporation method with real-time impedance monitoring to improve cell perforation efficiency and cell viability using a microelectrode array chip. The microchip contains a plurality of sextupole-electrode units patterned in an array, which are used to perform in situ electroporation and real-time impedance monitoring on single cells. The dynamic recovery processes of single cells under electroporation are tracked in real time via impedance measurement, which provide detailed transient cell states and facilitate understanding the whole recovery process at the level of single cells. We define single-cell impedance indicators to characterize cell perforation efficiency and cell viability, which are used to optimize electroporation. By applying the proposed electroporation method to different cell lines, including human cancer cell lines and normal human cell lines individually, optimum stimuli are determined for these cells, by which high transfection levels of enhanced green fluorescent protein (EGFP) plasmid into cells are achieved. The results validate the effectiveness of the proposed single-cell individualized electroporation/transfection method and demonstrate promising potential in applications of cell reprogramming, induced pluripotent stem cells, adoptive cell therapy, and intracellular drug delivery technology.

## Electroporation: Single-cell electroporation with real-time monitoring using a microelectrode chip

Electroporation involves applying an electrical field to cells to facilitate molecule delivery in therapeutics or diagnostics for personalized medicine, and a novel technique allows single-cell individualized electroporation with real-time monitoring using a microelectrode chip. Electroporation employs electrical stimulation to increase the permeability of cell membranes, but achieving high perforation efficiency while maintaining good cell viability remains a great challenge. A team headed by Rong Zhu at Tsinghua University, Beijing, China succeeded in developing an electroporation method at the single-cell level, offering improved cell perforation efficiency and cell viability by means of a microelectrode array chip. The technique allowed real-time monitoring of single-cell electroporation. The authors believe their system has considerable potential in various applications related to cell reprogramming, induced pluripotent stem cells, adoptive cell therapy, and intracellular drug delivery.

## Introduction

Cell transfection provides a powerful tool for cell reprogramming, induced pluripotent stem cells (iPSCs), adoptive cell therapy (ACT), and intracellular drug delivery technology^1–3^. Viral methods and chemical methods are commonly used for cell transfection. Viral methods typically use adeno-associated virus (AAV), retrovirus human immunodeficiency virus (HIV), and herpes simplex virus (HSV) to transfect exogenous genes. Chemical methods commonly use cationic lipids or polymers complexed with DNA to achieve transfection. However, these methods have limitations such as immune responses, unwanted mutagenesis and toxicity^4,5^. Physical methods, including microinjection^6^, ultrasound^7^, laser^8^, and electroporation (EP)^9^, have also been used to induce a transient opening of the cell membrane for transfection. Among physical methods, EP has been widely used in cell transfection because of its simplicity and easy integration^10^.

EP is a technique that utilizes electrical stimulation to induce permeability increases in cell membranes^11^. The conventional EP technique is bulk electroporation (BEP), which perforates the whole population of cells in an electric field of a few kilovolts per centimeter by applying hundreds or thousands of volts^12^. To study cell heterogeneity, single-cell EP has been proposed since the early 2000s by using carbon fibers^13^, micro-fabricated chips^14^, etc. Recently, researchers have explored various micro-device and nano-devices as effective means for achieving single-cell EP and decreasing EP voltage^15,16^, called micro-electroporation (MEP)^17^ or nano-electroporation (NEP)^18^. According to the architecture of the device, MEP entails microfluidic channel EP^19^, microcapillary EP^20^, microarray EP^21,22^, etc. In microfluidic channel EP, single cells flow through a microchannel and are perforated via electrodes on both ends of the channel^19^. It has high throughput but is difficult to track the response process of cell EP. Microcapillary EP uses a tip-type microcapillary device to allow selective EP on specific cells but is time consuming^20^. Microarray EP uses microwell array^21^ or microelectrode array^22^ to achieve high-throughput parallel transfection, but real-time monitoring of cell status is still a challenge. NEP devices include nanochannel EP^18^, nanostraw EP^23^, nanopillar EP^24^, nanoprobe (or nanofountain probe) EP^25^, nanoelectrode EP^26^, etc. NEP focuses the perforating electric field on a nanosized portion of the cell membrane, which enables a precise amount of drug or gene delivery^15,16^.

The major drawbacks of existing EP are substantial cell death caused by inappropriate electric stimuli and only partially successful membrane repair. Therefore, optimization of EP parameters is necessary to balance cell perforation efficiency and cell viability. Monitoring the dynamic process of EP and cellular recovery is one solution for this issue. However, the EP-induced pores are tiny and unstable. Therefore, they are difficult to visualize directly by optical microscopy and electron microscopy. Fluorescent dye is a way to assess EP in which light intensity represents the EP extent. However, the fluorescent method requires cells to be labeled via staining and can only reflect the transient state of cells rather than the dynamic recovery process. Comparatively, the electrical measurement approach is a simple, label-free and real-time method to monitor EP. Patch-clamp has been used to characterize single-cell EP, but it is time consuming and somewhat invasive to cells^27,28^. Electrical impedance spectroscopy (EIS) measures the electrical impedance of the cell, where a frequency-dependent signal is applied and the electrical responses are recorded. EIS technology is an effective method to study cells due to its simple, label-free, and easy integration^29^. Microfluidic impedance cytometry (MIC) makes single cells pass through a constricted microchannel to fix the cell shape and reduce leakage current, where the electrodes on both sides of the fluidic channel carry out EP and detect cell impedance^30^. However, the perforated cell is squeezed in the microchannel rather than its natural state, which may hinder cell recovery^31^. Electric cell-substrate impedance spectroscopy (ECIS) has been proposed to monitor the status of adherent cells by using microelectrodes on a substrate^32^.

We have developed a microelectrode array chip that integrates multiple functions of cell positioning, MEP, and impedance monitoring^33–36^. In this paper, we propose a single-cell individualized EP method via real-time impedance monitoring to balance perforation efficiency and cell viability using the microchip. By means of in situ impedance measurements, the dynamic response of a single cell to EP is monitored in real time, which facilitates the optimization of EP. We establish an analytical method of single-cell EP using the impedance indicators to characterize perforation efficiency and cell viability. By using the proposed impedance indicators, we optimize EP for various cell lines, including HeLa, MCF-7, and 293T, and achieve highly effective transfection of EGFP plasmid into these cells. The proposed single-cell individualized EP method can be also utilized to highly effective transfection for diverse cell lines and shows promising potential applications in personalized therapy and diagnosis.

## Results and discussions

### System and microchip

**Fig. 1:**
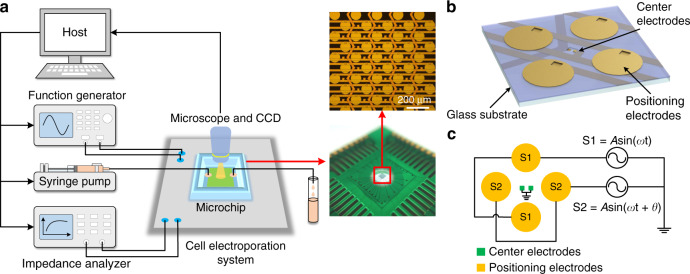
Schematic diagram of the system and microchip. **a** Sketch of the electroporation system with a microelectrode array chip. **b** Schematic view of the microelectrode unit on the chip. **c** Signal application mode for cell positioning.

An EP system with a microelectrode array chip is shown in Fig. 1a. It comprised a platform to transmit control signals onto the microchip, which was mounted on a microscope (DM2500, Leica) with a CCD camera (DFC450C, Leica). A function generator (AFG3250, Tektronix) was used to generate electric signals for cell positioning and cell EP. An impedance analyzer (PARSTAT 4000, Princeton Applied Research) was used to perform impedance measurement. A syringe pump (ExiGo pump, Cellix) was used to inject fluid sample into the microchip. The function generator, impedance analyzer, and syringe pump were all controlled by a host. The inset of Fig. 1a shows the prototype of the microchip.

In Fig. 1b, a schematic view of the microelectrode unit on the chip is illustrated. The microchip consisted of a plurality of sextupole-electrode units patterned in an array. In each unit, there were a pair of center microelectrodes and quadrupole positioning electrodes^33–35^. The center microelectrodes were used to perform in situ cellular EP and real-time impedance monitoring. The quadrupole positioning electrodes were used to trap and position live cells suspended in culture medium onto the unit center based on negative dielectrophoresis (nDEP)^36–38^. The dimensions of the center microelectrodes were 7 × 7 μm^2^, and the gap between them was 7 μm, which allowed single-cell EP and measurement. The quadrupole positioning electrodes were designed with 100 μm diameters and 100 μm gaps. The fabrication process of the microchip is described in Supplementary Note 1. Three titanium and gold (Ti/Au) layers were patterned to form leads and electrodes through a lift-off process on a glass wafer. Two SiO_2_ insulating layers were deposited between the three metal layers by plasma-enhanced chemical vapor deposition (PECVD). Insulating layers were etched to expose windows for connecting electrodes and leads. The microchip was mounted on a printing circuit board (PCB) with wire bonding. A polydimethylsiloxane (PDMS) pool was adhered onto the PCB board around the microchip to form a cell sample pool. Figure 1c illustrates the signal application mode for cell positioning based on nDEP. Two sinusoidal signals were applied onto two pairs of opposite positioning electrodes, while the center electrodes were grounded. Live cells were trapped and manipulated in each unit by modulating the phase difference *θ* of the two sinusoidal signals.

### Experimental procedure

**Fig. 2:**
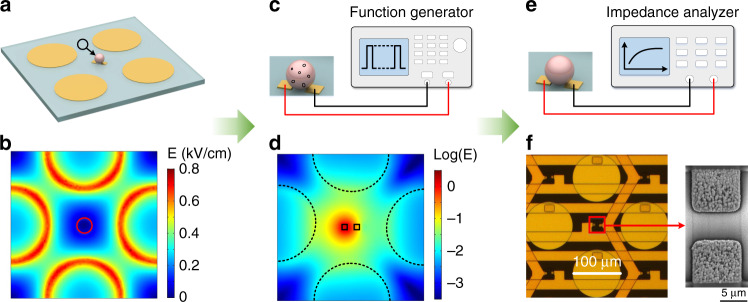
Experimental procedure for cell positioning, electroporation, and impedance measurement. **a** Schematic view of single-cell positioning at the unit center by nDEP forces. **b** Simulation result of electric field intensity on a plane 5 μm above the microchip surface. The signal phase difference was 180°, the peak-to-peak voltage was 2.8 V_pp_, and the frequency was 100 kHz. The red circle marks the region with the minimum electric field intensity. **c** Electric pulses were applied to the center electrodes for cell electroporation. **d** Simulation result of the electric field intensity at a voltage of 6 V. **e** The impedance measurement was conducted to monitor the cellular recovery process. **f** A scanning electron microscope (SEM) image of the surface morphology of the center microelectrodes.

Cell samples (cells in culture medium) were pumped into the sample pool of the microchip. After ~1 min, the cell sample became stable. For cell positioning, two sinusoidal signals with a peak-to-peak voltage of 2.8 V_pp_, a frequency of 100 kHz and a phase difference of 180° were applied to the positioning electrodes, while the center electrodes were grounded (Figs. 2a and 1c). Cells were moved into the unit center that had the minimum electric field intensity by nDEP forces (red circle in Fig. 2b). The cell positioning process could be completed within 30 s (Supplementary Video 1). A detailed description of nDEP-based cell positioning is shown in Supplementary Note 2.

After cell positioning onto the center electrodes, cells were cultured for 4 h to adhere onto the substrate. Before EP, the solution was exchanged with EP buffer by a syringe pump. Then, EP was conducted by applying electric pulses to the center electrodes while the positioning electrodes were grounded (Fig. 2c). Figure 2d shows the distribution of the electric field intensity under a voltage of 6 V, where a high electric field was located at the center area. After cell EP, single-cell impedance measurements were conducted to monitor the cellular recovery process by using the center microelectrodes and an impedance analyzer (Fig. 2e). After impedance measurements were acquired, the solution was exchanged to the culture medium by a syringe pump to maintain cell viability. To enhance the sensitivity of the single-cell impedance measurement, the surfaces of the center microelectrodes were modified with gold nanostructures to enlarge the effective surface area and reduce the double-layer impedance existing in the electrode–electrolyte interface. The detailed fabrication process of surface modification is described in Supplementary Note 3 and our previous article^34^. Figure 2f shows the gold nanostructures on the center microelectrodes taken with a scanning electron microscope (Gemini SEM500, Zeiss, Germany). Single-cell impedance measurements in every unit of array on the microchip were accessed and scanned by using an addressing method (Supplementary Note 4).

### Impedance monitoring during the cell recovery process after EP

EP is a physical transfection method that uses electrical pulses to create temporary pores in cell membranes, which enables substances such as nucleic acids to enter cells. It is a highly efficient strategy for the introduction of foreign nucleic acids into various cell lines. During EP, the parameters of imposed electric stimuli (pulse amplitude, pulse number, pulse width, and pulse frequency) are very important conditions for achieving high perforation efficiency and good cell viability. Because of the diversity of cell lines and cell heterogeneity, single-cell individualized EP with appropriate stimulus parameters is imperative. Optimization of EP aims to find specific electric parameters to maximize perforation efficiency while maintaining good cell viability. To achieve this goal, understanding the cell dynamic recovery process after EP is necessary.

**Fig. 3:**
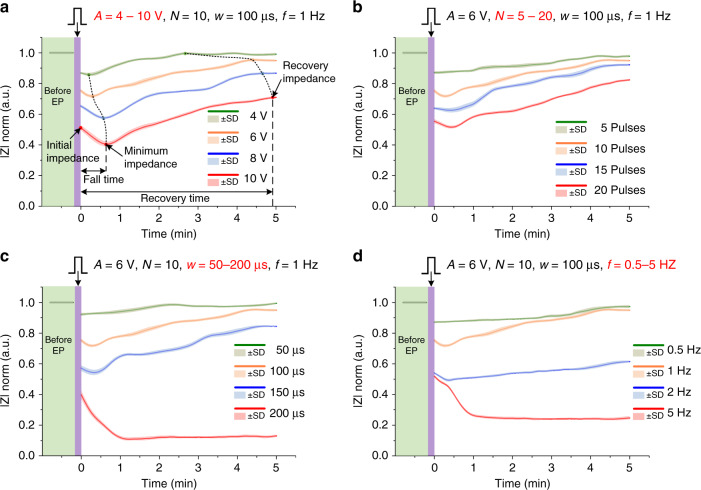
Impedance measurement (at 100 kHz) of single HeLa cells in the recovery process under different electroporation parameters. **a** The pulse amplitude (denoted as *A*) changed from 4 to 10 V, while the pulse number (*N* = 10), pulse width (*w* = 100 μs), and pulse frequency (*f* = 1 Hz) were fixed. **b** The pulse number (denoted as *N*) changed from 5 to 20, while the pulse amplitude (*A* = 6 V), pulse width (*w* = 100 μs) and pulse frequency (*f* = 1 Hz) were fixed. **c** The pulse width (denoted as *w*) changed from 50 to 200 μs, while the pulse amplitude (*A* = 6 V), pulse number (*N* = 10), and pulse frequency (*f* = 1 Hz) were fixed. **d** The pulse frequency (denoted as *f*) changed from 0.5 to 5 Hz, while the pulse amplitude (*A* = 6 V), pulse number (*N* = 10), and pulse width (*w* = 100 μs) were fixed.

Figure 3 shows the impedance measurement (at 100 kHz) of a single HeLa cell in its recovery process under different EP parameters. The impedances were normalized by taking the impedance before EP as 1 and the impedance without cells as 0. Mean values ± standard deviations (SDs) were estimated from at least five single-cell measurements. There are four main parameters of EP signals: pulse amplitude (denoted as *A*), pulse number (denoted as *N*), pulse width (denoted as *w*) and pulse frequency (denoted as *f*). In Fig. 3a, the pulse amplitude changed from 4 to 10 V, while the pulse number (*N* = 10), pulse width (*w* = 100 μs), and pulse frequency (*f* = 1 Hz) were fixed. Figure 3b–d shows the changes in pulse number (*N*), pulse width (*w*), and pulse frequency (*f*), while the other parameters were unchanged. We conducted contrast experiment of impedance measurements under different EP parameters with and without cells on the microelectrodes. The results (Supplementary Note 5) indicated that background impedance without cells remained steady before and after EP. Cell impedance without EP (Supplementary Note 6) remained steady as well. These results indicated that the impedance change was entirely caused by the cellular response to EP. In other words, the impedance signal could clearly indicate the cellular response to EP.

To facilitate qualitative analysis, we defined some impedance indicators, including the initial value of cell impedance after EP as the initial impedance (time = 0), the minimum value of cell impedance after EP as the minimum impedance, the value when the impedance reached steady state as the recovery impedance, the period time from the initial impedance to the minimum impedance as the fall time, and the period time from the initial impedance to the recovery impedance as the recovery time. The following phenomena were observed according to Fig. 3. The initial impedance was lower than the impedance before EP. This was due to the generation of cell membrane holes and the change in cell electrical properties caused by EP^9^. After EP, impedance first decreased for a short time (fall time) and reached the minimum impedance. We supposed that this phenomenon was due to the accompanying effect of electrical stimulation, such as cellular metabolic product accumulation or transient osmotic imbalance^39,40^. Afterwards, impedance increased gradually and reached recovery impedance in ~200–300 s (recovery time), which was caused by the recovery of cell electrical properties^41,42^. However, recovery impedance was still lower than the impedance before EP. Finally, the impedance reached a stable stage. Under different stimuli, the response processes of impedance changes were different. When the EP pulses were weak, cell impedance did not decline but directly rose instead, as shown by the green curves in Fig. 3b–d. When the EP pulses were very strong, cell impedance declined greatly, eventually stabilized at the minimum impedance, and the recovery process was terminated, as shown by red curves in Fig. 3c, d, which indicated irreversible EP and cell death. In contrast, other curves in Fig. 3 indicated reversible EP, where the values of initial impedance, minimum impedance, and recovery impedance decreased with increasing the pulse intensity (pulse amplitude, number, width, and frequency), while the fall time and recovery time increased correspondingly.

### Characterizing EP with impedance indicators

The above impedance indicators could be used not only to analyze the recovery process after EP but also to characterize perforation efficiency and cell viability. Since the initial descent impedance defined as the difference between cellular impedance before EP and its initial impedance after EP was mainly affected by cell membrane pores and cell membrane permeability, it could be used to characterize perforation efficiency. The larger the initial descent impedance was, the higher the perforation efficiency became. On the other hand, cell impedance recovery signified restoration of cell viability. Therefore, recovery impedance could be used to characterize cell viability after EP. The larger the recovery impedance was, the more viable the cell became. The increases in pulse amplitude, number, width, and frequency could enhance perforation efficiency but reduce cell viability. Among the four EP parameters, pulse width and pulse frequency had the greatest influence on cells. Cell viability decreased rapidly with increasing pulse width and pulse frequency. That is, the cell could not tolerate pulse signal with long width and high frequency. The experiments showed that the pulse width of 100 μs and the pulse frequency of 1 Hz were appropriate parameters for most cells.

**Fig. 4:**
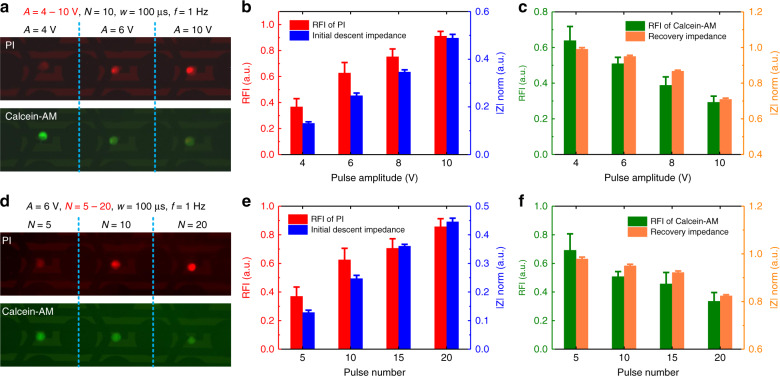
Comparative analyses of fluorescent staining results and impedance indicators for electroporation under different condition. **a** Fluorescent images of PI and Calcein-AM for HeLa cell electroporation at different pulse amplitudes. **b** Relative fluorescence intensity (RFI) of PI and initial descent impedance at different pulse amplitudes. **c** Relative fluorescence intensity of Calcein-AM and recovery impedance at different pulse amplitudes. **d** Fluorescent images of PI and Calcein-AM for HeLa cell electroporation for different pulse numbers. **e** Relative fluorescence intensity of PI and initial descent impedance for different pulse numbers. **f** Relative fluorescence intensity of Calcein-AM and recovery impedance for different pulse numbers. Error bars indicate the standard deviation (SD).

To verify the above impedance indicators in characterizing cell perforation efficiency and cell viability, we performed a comparative analysis using the fluorescence method. Figure 4a shows the fluorescent images of propidium lodide (PI) and Calcein-AM for HeLa cell EP under different pulse amplitudes. Pulse signals applied on the cells had pulse amplitudes (*A*) of 4, 6, and 10 V; a pulse number (*N*) of 10; a pulse width (*w*) of 100 μs; and a pulse frequency (*f*) of 1 Hz. With increasing pulse amplitude, the red fluorescence of PI gradually strengthened, which indicated an increase in perforation efficiency. However, the green fluorescence of Calcein-AM gradually weakened, which implied a decrease in cell viability.

To compare the fluorescence results with impedance indicators, Fig. 4b shows the relative fluorescence intensity (RFI) of PI and initial descent impedance under different pulse amplitudes. Mean values and SDs of RFI were calculated from the measurements of fluorescent images shown in Supplementary Note 7. The initial descent impedance in Fig. 4b was extracted from Fig. 3a. As the pulse amplitude increased, both the RFI of PI and the initial descent impedance increased. The results indicated that there was a positive correlation between the initial descent impedance and perforation efficiency. The higher the perforation efficiency was, the greater the initial descent impedance became, which corresponded to a lower initial impedance. In addition, Fig. 4c shows that the relative fluorescence intensity (RFI) of Calcein-AM was correlated with the recovery impedance at different pulse amplitudes. With the increase in pulse amplitude, both the RFI of Calcein-AM and recovery impedance decreased. The above analysis indicated that the level of recovery impedance reflected the cell viability. The better the cell viability was, the greater the recovery impedance was.

Figure 4d shows the fluorescent images of PI and Calcein-AM for single HeLa cells perforated under different pulse numbers. Pulse signals applied on the cells had a pulse amplitude (*A*) of 6 V; pulse numbers (*N*) of 5, 10, and 20; a pulse width (*w*) of 100 μs; and a pulse frequency (*f*) of 1 Hz. The increase in pulse number resulted in enhanced red fluorescence intensity of PI and reduced green fluorescence intensity of Calcein-AM, which indicated an increase in perforation efficiency and a decrease in cell viability. Figure 4e compares the RFI of PI and initial descent impedance for different pulse numbers. Figure 4f compares the RFI of Calcein-AM and recovery impedance for different pulse numbers. The results verified that initial descent impedance was positively correlated with perforation efficiency, and recovery impedance was positively correlated with cell viability.

As mentioned above, good EP is associated with maximizing perforation efficiency while maintaining good cell viability. That is, both the initial descent impedance and the recovery impedance are as large as possible. According to the results shown in Fig. 4 and Supplementary Note 7, we proposed an optimization criteria that good EP required an initial descent impedance above 0.25 and a recovery impedance above 0.9. For HeLa cells, the optimized EP parameters were determined to be a pulse amplitude of 6 V, pulse numbers of 10–15, a pulse width of 100 μs and a pulse frequency of 1 Hz based on the above criteria. We further extended this optimization criteria to other cell lines.

**Fig. 5:**
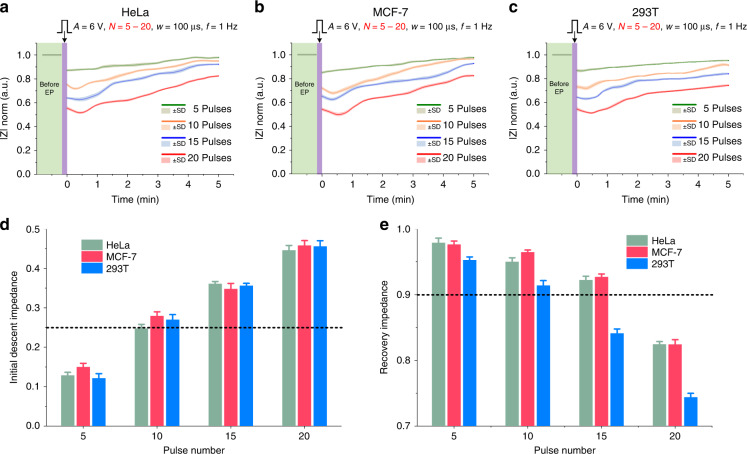
Impedance monitoring results of different cell lines after electroporation. **a**–**c** The impedance of HeLa, MCF-7, and 293T cells after electroporation with different pulse numbers. **d** The initial descent impedance varied with the pulse number. **e** Recovery impedance varied with the pulse number. Error bars indicate the standard deviation (SD).

We applied EP to various cell lines, including human cancer cells (HeLa, MCF-7) and normal human cells (293T), using pulse numbers (*N*) of 5–20, a pulse amplitude (*A*) of 6 V, a pulse width (*w*) of 100 μs, and a pulse frequency (*f*) of 1 Hz. The single-cell impedance monitoring results for these cells are shown in Fig. 5. Mean values ± standard deviations (SDs) were estimated from at least five single-cell measurements. The impedance recovery laws of these cell lines were similar to those represented in Fig. 3. Figure 5d, e show the initial descent impedances and recovery impedances of these cell lines under EP with different pulse numbers, respectively. The initial descent impedances of these cell lines under the same EP conditions were almost the same, which indicated that different cell lines had similar perforation efficiencies under the same EP signal. Regarding recovery impedance, HeLa and MCF-7 cells had similar values, and both had higher values than that for 293T cells. This was because both HeLa and MCF-7 cells were human cancer cell lines, but 293T cells were normal human cell line. Normal cell lines might have lower tolerances to electric stimulation than cancer cell lines^41^.

Based on the proposed optimization criteria, that is good EP requires an initial descent impedance above 0.25 (high perforation efficiency) and a recovery impedance above 0.9 (high cell viability), we optimized EP for HeLa, MCF-7, and 293T cells. According to Fig. 5, the optimum EP parameters for HeLa and MCF-7 cells were determined to be a pulse amplitude of 6 V, pulse numbers of 10–15, a pulse width of 100 μs and a pulse frequency of 1 Hz. For 293T cells, the optimum EP parameters were determined to be a pulse amplitude of 6 V, a pulse number of 10, a pulse width of 100 μs and a pulse frequency of 1 Hz.

### Plasmid transfection by EP

To verify the effectiveness of the above optimized EP parameters, we performed EGFP plasmid transfection experiments on HeLa, MCF-7, and 293T cells. We analyzed three experimental groups: the control group, EP group 1 (EG1), and EP group 2 (EG2). The control group refers to cells cultured with EGFP plasmid but without EP. The EP parameters of the EG1 and EG2 groups were selected from the above optimized EP parameters. The EP signal of the EG1 group entailed a pulse amplitude of 6 V, a pulse number of 10, a pulse width of 100 μs and a pulse frequency of 1 Hz. The EP signal of EG2 entailed a pulse amplitude of 6 V, a pulse number of 15, a pulse width of 100 μs and a pulse frequency of 1 Hz. Both EG1 and EG2 groups satisfied the optimization criteria for HeLa and MCF-7 cells, and only EG1 satisfied the optimization criteria for 293T cells.

**Fig. 6:**
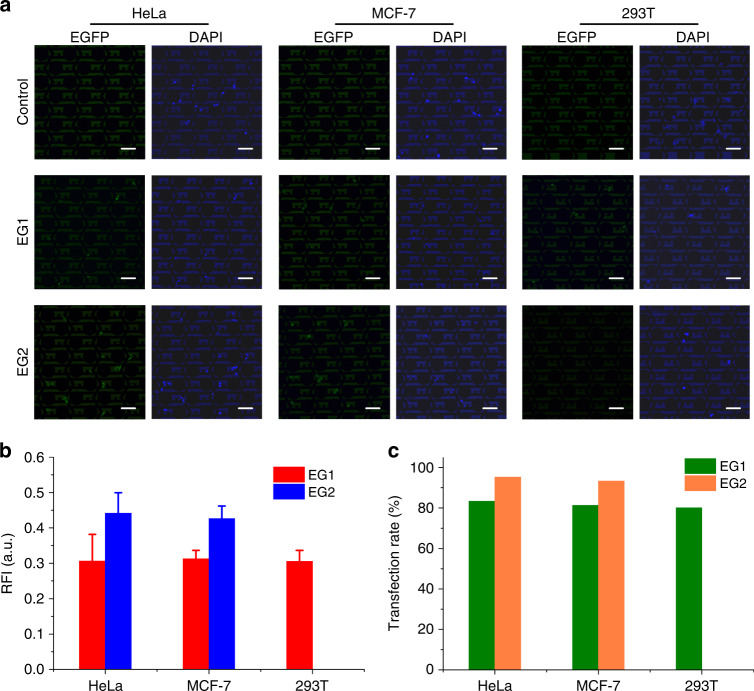
EGFP plasmid transfection results of HeLa, MCF-7, and 293T cells. **a** Fluorescence micrographs of EGFP and DAPI: EG1 experimental group one, EG2 experimental group two (scale bar = 100 μm). **b** Relative fluorescence intensity (RFI) of the EGFP plasmid expressed in cells. Error bars indicate standard deviations. **c** Transfection rates of the EGFP plasmid.

Figure 6a shows the fluorescence results of HeLa, MCF-7, and 293T cells. Green fluorescence represents EGFP plasmid expression. Blue fluorescence represents the staining of nuclei by DAPI. A few cells slightly deviated from the center electrodes due to cell migration and fluid exchange. The cells of the control groups did not emit green fluorescence, which indicated that the EGFP plasmid could not enter the cells autonomously. In the EP groups (EG1 and EG2) of HeLa and MCF-7 cells, the cells in the center area of the electrode units emitted green fluorescence, which indicated that good plasmid transfection was achieved for these two cell lines. For 293T cells, the transfection results shown in Fig. 6a indicated that the EG1 group of 293T cells achieved good plasmid transfection. Most cells were successfully transfected and emitted green fluorescence. The EP signal for EG2 group containing more pulses would damage 293T cells, and most of the cells died and floated away. The experimental result again verified the effectiveness of the above EP optimization. Enlarged images of the EGFP transfection results are shown in Supplementary Note 8.

Figure 6b shows the RFI of the EGFP plasmid expressed in cells. The RFI of the EGFP plasmid of the EG1 group with a pulse number of 10 was almost the same for the three cell lines, which was consistent with the results shown in Fig. 5d, e. The reason was that the optimization criteria (the initial descent impedance above 0.25 and the recovery impedance above 0.9) were satisfied for all three cell lines. In other words, the EP parameters of the EG1 group were good for all cell lines. The RFI of the EG2 group was higher than that of the EG1 group for HeLa and MCF-7 cells but invalid for 297T cells. For cancer cells, the EP pulse number could be increased from 10 to 15 (i.e. from EG1 to EG2) to improve the EP perforation efficiency, which was consistent with the increase in the initial descent impedance shown in Fig. 5d. EG2 EP parameters with a pulse number of 15 was unfavorable for 293T cells because cell viability was seriously damaged, as shown in Fig. 5e, and the recovery impedance was <0.9.

Figure 6c shows the transfection rates of the EGFP plasmid. The transfection rates of all cell lines in the EG1 group reached 80–85%. The transfection rates of HeLa and MCF-7 cells in the EG2 group reached ~95%. High transfection efficiencies further validated the effectiveness of the proposed EP optimization method.

## Conclusion

In this paper, we proposed a single-cell individualized EP method via real-time single-cell impedance monitoring using a microelectrode array chip. The microchip contained an array of sextupole-electrode units, which were used for cell positioning, in situ EP, and real-time impedance measurement. Single-cell impedance measurements were used to track the cellular dynamic response to EP in real time. We proposed single-cell impedance indicators to characterize perforation efficiency and cell viability. The initial descent impedance was positively related to perforation efficiency, and recovery impedance was positively related to cell viability. Optimization criteria was established, that is good EP required an initial descent impedance >0.25 (high perforation efficiency) and a recovery impedance >0.9 (high cell viability). Based on this criteria, we optimized the EP parameters for different cell lines, including HeLa, MCF-7, and 293T cells. The optimized EP parameters for HeLa and MCF-7 cells were a pulse amplitude of 6 V, pulse numbers of 10–15, a pulse width of 100 μs and a pulse frequency of 1 Hz. For 293T cells, the optimized EP parameters were a pulse amplitude of 6 V, a pulse number of 10, a pulse width of 100 μs, and a pulse frequency of 1 Hz. Furthermore, we transfected the EGFP plasmid into individual HeLa, MCF-7, and 293T cells by using the optimized EP parameters and achieved a high transfection efficiency above 80% for 293T cells and transfection efficiencies above 95% for HeLa and MCF-7 cells. The experiments proved the effectiveness of the EP method. The proposed single-cell individualized EP method could be extended to highly efficient gene transfections for diverse cell lines and also demonstrated promising application potential in cell reprogramming, iPSCs, ACT, and intracellular drug delivery technology.

## Materials and methods

### Cells and chemicals

HeLa (the human epithelioid cervix carcinoma cell line), MCF-7 (the human breast cancer cell line), and 293T (the human embryonic kidney cell line) cells were cultured as a monolayer in a 25 cm^2^ culture flask containing Dulbecco’s modified Eagle medium (DMEM) supplemented with 10% fetal bovine serum (FBS), 100 units/mL penicillin and 100 μg/mL streptomycin at 37 °C in a 5% CO_2_ atmosphere. Before EP, the cells were harvested by 0.25% trypsin/EDTA and then suspended in culture medium at a concentration of 2 × 10^5^ cells/mL.

The EP buffer contained 10 mM NaCl, 1.7 mM MgCl_2_, 100 mM sorbitol, and 10 mM HEPES (pH adjusted to 7.4 at 25 °C).

### Fluorescent staining

PI and Calcein-AM were used to assess perforation efficiency and cell viability, respectively. PI is a membrane impermeable dye without auto fluorescence. When applying EP to cells, PI penetrates the cell membrane, binds to nucleic acids inside the cell and fluoresces red. Therefore, the fluorescence intensity of PI was used to assess perforation efficiency^43,44^. Calcein-AM is initially non-fluorescent and can passively diffuse across the cell membrane. After entering a live cell, enzymes in the cytoplasm decompose Calcein-AM molecule, resulting in green fluorescence. Good cell viability corresponds to strong green fluorescence in the presence of Calcein-AM. Therefore, Calcein-AM is commonly used as an indicator of cell viability after EP^43^. In fluorescence measurement experiments, 5 μg/mL PI (Sigma Chem Co., USA) was added to EP buffer before EP. After EP, 2 μmol/L Calcein-AM (Sigma Chem Co., USA) was added to the EP buffer. After 5 min of incubation, the cells were observed by fluorescence microscopy.

### EGFP plasmid transfection

Single cells were suspended in the culture medium and poured into the microchip. The cells were then positioned at the unit centers of the electrode array by nDEP manipulation and were cultured for 4 h to adhere onto the substrate. Before EP, the solution was exchanged with EP buffer containing 10 µg/mL EGFP plasmid (VectorBuilder, China). Then, the specified EP signals were applied to the cells. After that, EP buffer was exchanged to the culture media for subsequent cell culture. After 24 h, the results of plasmid transfection were observed using a fluorescence microscope. The nuclei were stained with DAPI (Macgene, China) and observed under a fluorescence microscope. The gene map of the EGFP plasmid is shown in Supplementary Note 9.

## Supplementary information

Supplementary Information

Supplementary Video 1
