# Supplementary material for: Single-cell individualized electroporation with real-time impedance monitoring using a microelectrode array chip
Source: Microsyst Nanoeng. 2020 Oct 19;6:81. doi: 10.1038/s41378-020-00196-0 (PMC8433324; doi:10.1038/s41378-020-00196-0)
Supplement: Supplementary file 1 — Supplementary Information [file 41378_2020_196_MOESM1_ESM.docx]

Supplementary Information for

Single-cell individualized electroporation with real-time impedance monitoring using microelectrode array chip

Zhizhong Zhang, Tianyang Zheng, and Rong Zhu*

**Corresponding Author:**

Dr. & Prof. Rong Zhu

Department of Precision Instrument, Tsinghua University, Beijing 100084, China
E-mail: zr_gloria@mail.tsinghua.edu.cn

Tel: +86-010-62788935

**Table of Content**

- Fabrication of the microchip
- nDEP-based cell positioning
- Process of electrode modification
- Addressing method for array chip
- Electroporation impedance with and without cells
- Impedance of cells without electroporation
- Fluorescence images of HeLa cell electroporation
- Enlarged images of the EGFP transfection results
- The gene map of the EGFP plasmid
- Supplementary Video

**Note 1. Fabricationofthe microchip**


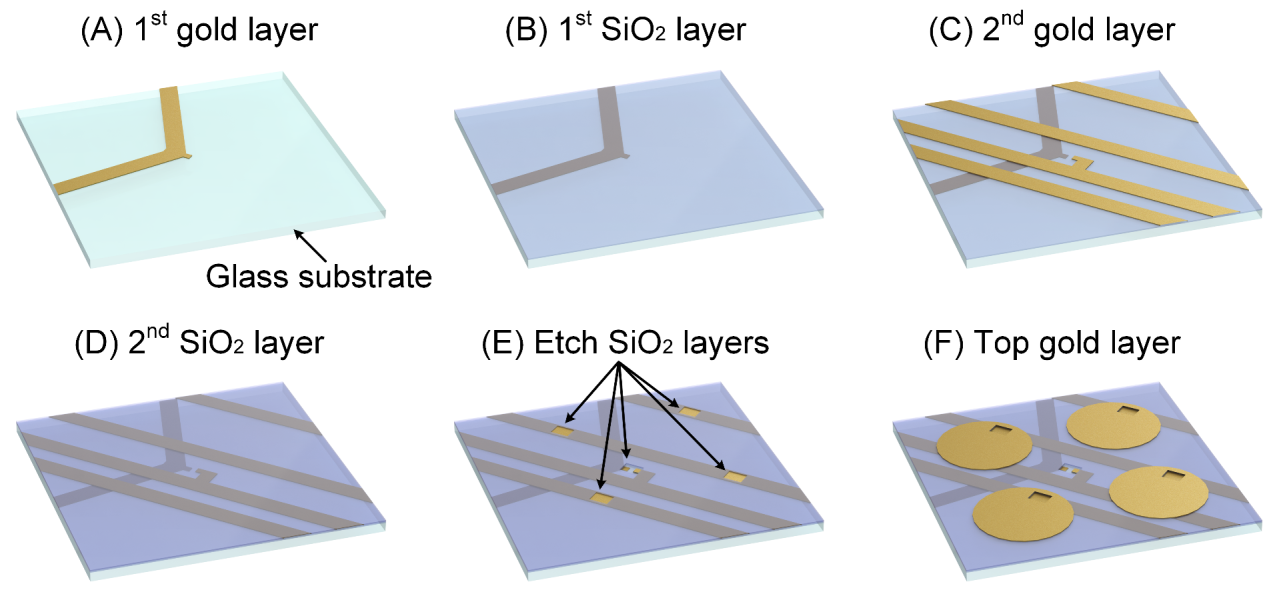


**Figure S1.** Fabrication processes of the microchip. (A) The 1^st^ gold layer was patterned on a glass wafer. (B) The 1^st^ SiO_2_ layer was deposited by PECVD. (C) The 2^nd^ gold layer was patterned through alift-off process. (D) The 2^nd^ SiO_2_ layer was deposited onthe chip. (E) The two SiO_2_ layers were selectively etched. (F) The top gold layer was patterned to form he positioning electrodes.

**Note 2. nDEP-based cell positioning**

A DEP force acting on a cell suspending in a fluidis given by^1^

$F_{DEP}=2\pi R^{3}\varepsilon_{m}\mathrm{Re}(f_{CM})\nabla\left(E^{2}\right)$ (S1)

where *R* is the cell radius, *ε_m_* is the medium permittivity, *E* is the electric field intensity, is the gradient operator, and Re(·) stands for the real part ofa complex variable. *f_CM_* is the Clausius-Mossotti (CM) factor. The CM factor is a complex number andis given by ^2^

$f_{CM}=\frac{\varepsilon_{c}^{*}-\varepsilon_{m}^{*}}{\varepsilon_{c}^{*}+2\varepsilon_{m}^{*}}$(S2)

$\varepsilon_{c}^{\text{*}}=\frac{C_{mem}^{*}R\varepsilon_{cyto}^{*}}{C_{mem}^{*}R+\varepsilon_{cyto}^{*}}$ (S3)

where $\varepsilon_{m}^{*}=\varepsilon_{m}-j\frac{\sigma_{m}}{\omega}$, $\varepsilon_{cyto}^{*}=\varepsilon_{cyto}-j\frac{\sigma_{cyto}}{\omega}$, $C_{mem}^{*}=C_{mem}-j\frac{G_{mem}}{\omega}$, *ω* is the angular frequency of the applied signal, and *j* is an imaginary unit, the meaning and value of other symbols are shown in Table 1.The sign of the real part of the CM factor determines the direction of the DEP force. If it is negative, the particles move toward low electric field region (negative DEP). If it is positive, the particles move toward high electric field region (positive DEP).From figure S2(A), the cell is affected by nDEP in the full frequency range. Figure S2(B) shows the result ofHeLa cell positioning.


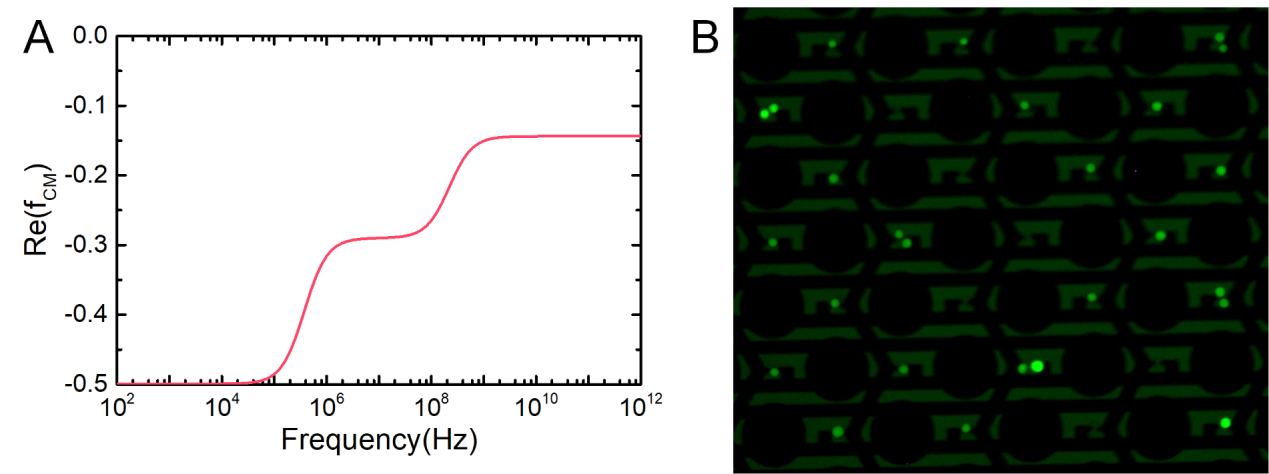


**Figure S2.**(A) The plot of the real part of the HeLa cell CM factor against the applied frequency. (B) The result ofHeLa cell positioning.

TableS1 Parameters of culture medium and HeLa cell^3^

| [Classify](file:///C:\Users\zzz\AppData\Local\youdao\dict\Application\7.2.0.0511\resultui\dict\?keyword=classify) | Parameters | Definition | Value | Unit |
| --- | --- | --- | --- | --- |
| Culture medium | *ε_0_* | Vacuum permittivity | 8.85×10^-12^ | *F/m* |
|  | *ε_m_* | Medium permittivity | 78*ε_0_* | *F/m* |
|  | *σ_m_* | Medium conductivity | 1.1 | *S/m* |
|  | *η* | Viscosity coefficient of medium | 1.003×10^-3^ | *Pa·s* |
| Cells | *ε_cyto_* | Cytoplasm permittivity | 47.5*ε_0_* | *F/m* |
|  | *σ_cyto_* | Cytoplasm conductivity | 0.36 | *S/m* |
|  | *C_mem_* | Specific membrane capacitance | 13.11 | *μF/cm^2^* |
|  | *G_mem_* | Specific membrane conductance | 100 | *S/m^2^* |
|  | $R$ | Cell radius | 7.0 | *μm* |
|  | *ρ* | Cell density | 1×10^3^ | *kg/m^3^* |

**References**

(1) Pohl, H. *Cambridge University Press, Cambridge***1978**.

(2) Jones, T. B.; Jones, T. B. *Electromechanics of particles*; Cambridge University Press, 2005.

(3) Huang, L.; Zhao, P.; Wang, W. *Lab Chip***2018**, *18*, 2359-2368.

**Note 3. Processof electrode modification**

**
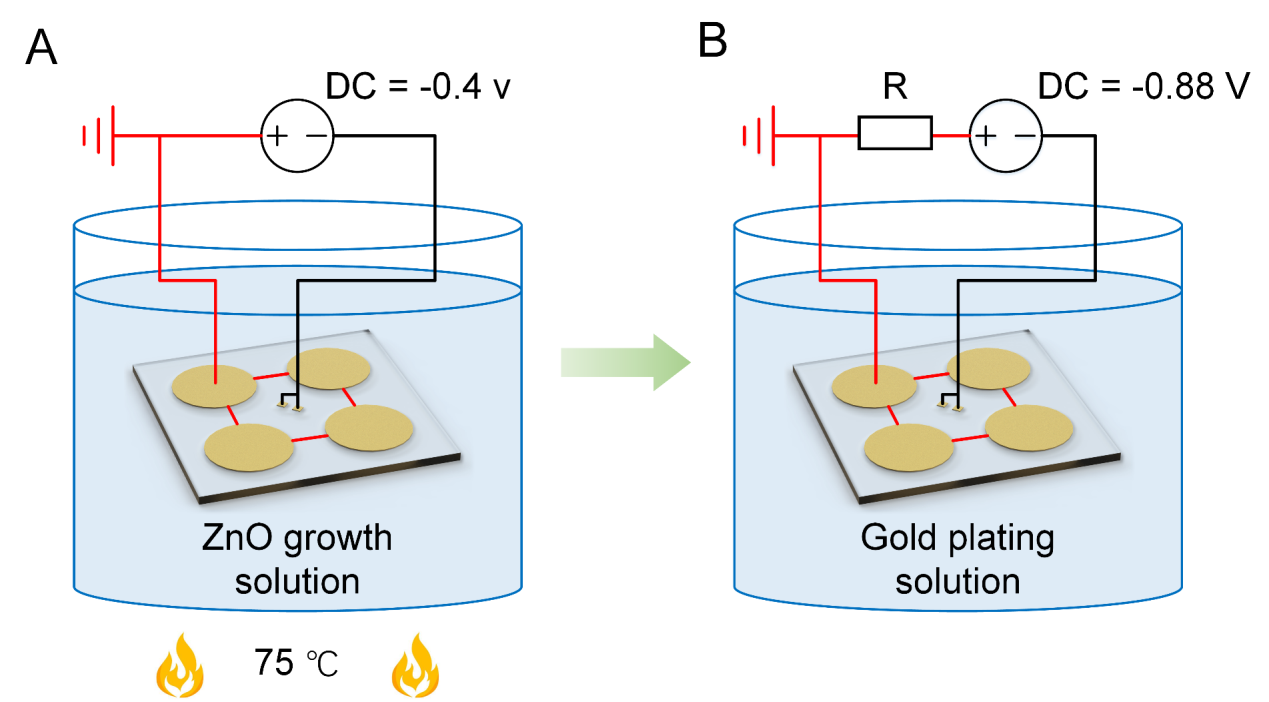
**

**FigureS3.**Process of electrode modification (A)The microchip was immersed in an ZnO growth solution at 75 °C for 1 hour. A negative voltageof −0.4 V was applied to the measuring electrodes and thepositioning electrodes were grounded. (B) The microchip was immersed in a gold platingsolution. A negative voltageof -0.88 V was applied to the measuring electrodes and the positioningelectrodes were grounded. The current limiting resistor R is 200 kΩ.

**Note 4. Addressing method for array chip**

**
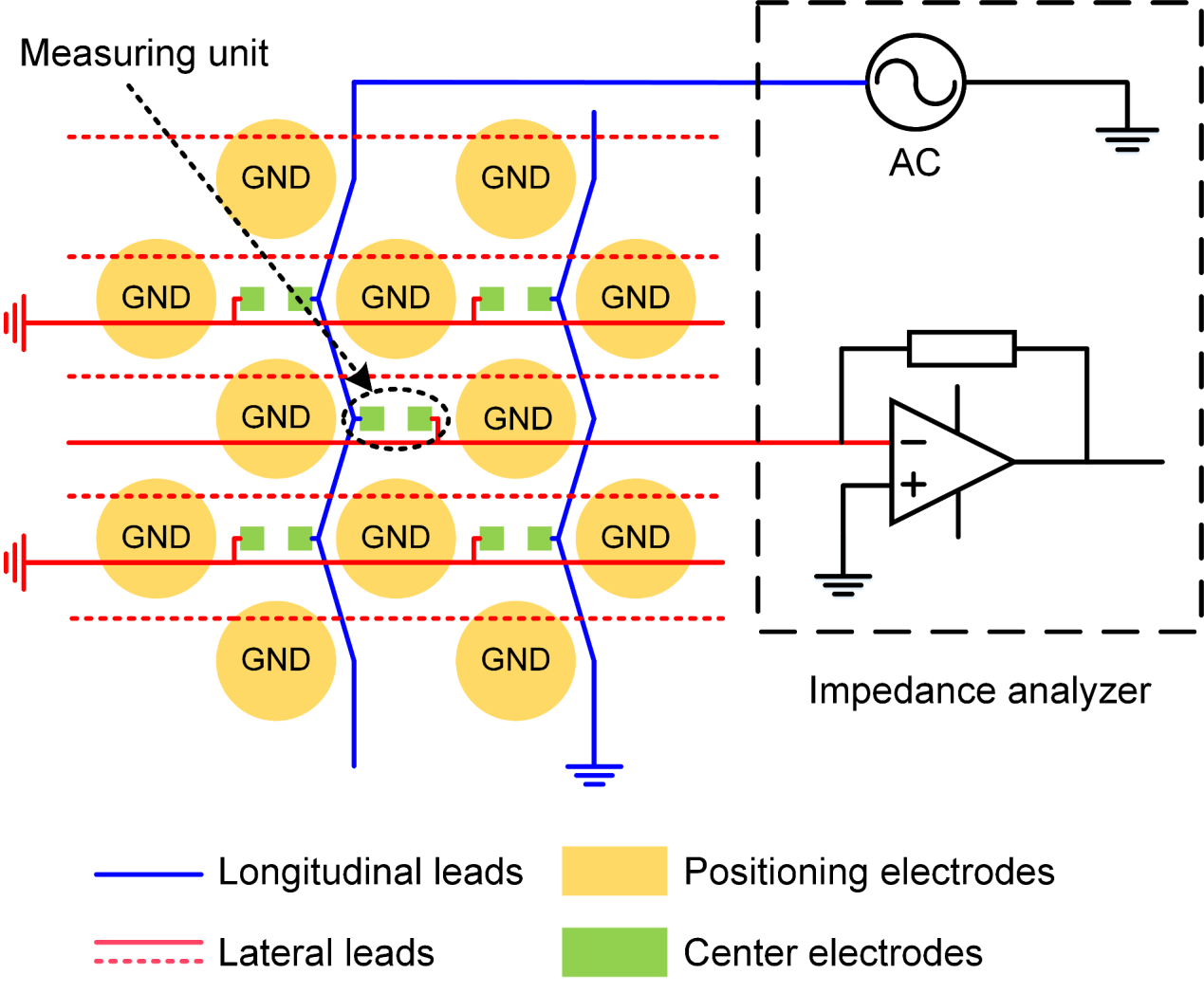
**

**Figure S4.** Schematic diagram of addressing method for array chip. The longitudinal leads and lateral leads of the center electrodes are connected to external addressing circuit, while the positioning electrodes are grounded. The impedance measurement at every unit could be accessed by addressing.

**Note 5. Electroporation impedancewith and without cells**

**
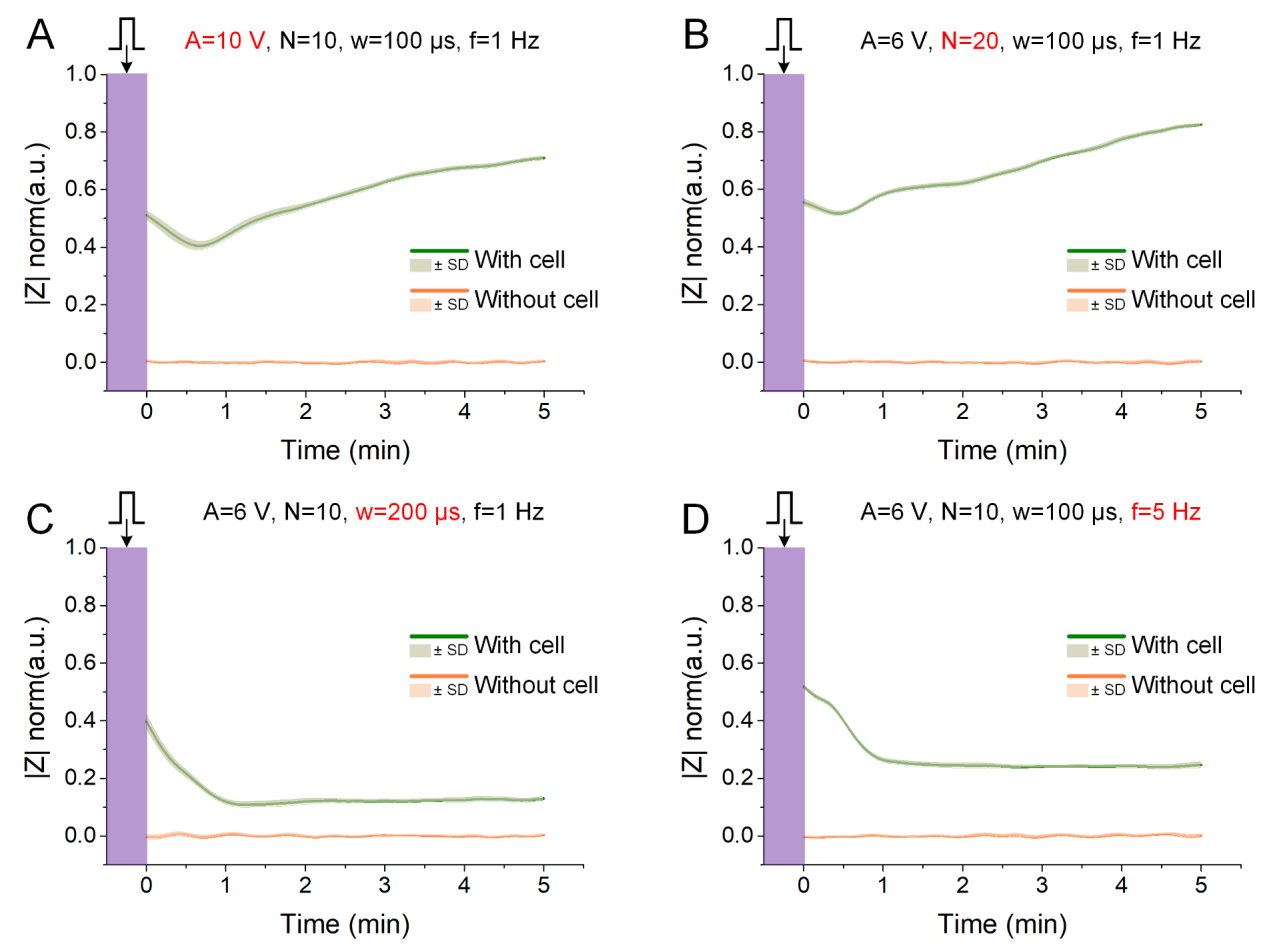
**

**Figure S5.** Electroporation impedance with and without cells under different electroporation parameters.

**Note 6. Impedance of cells without electroporation**


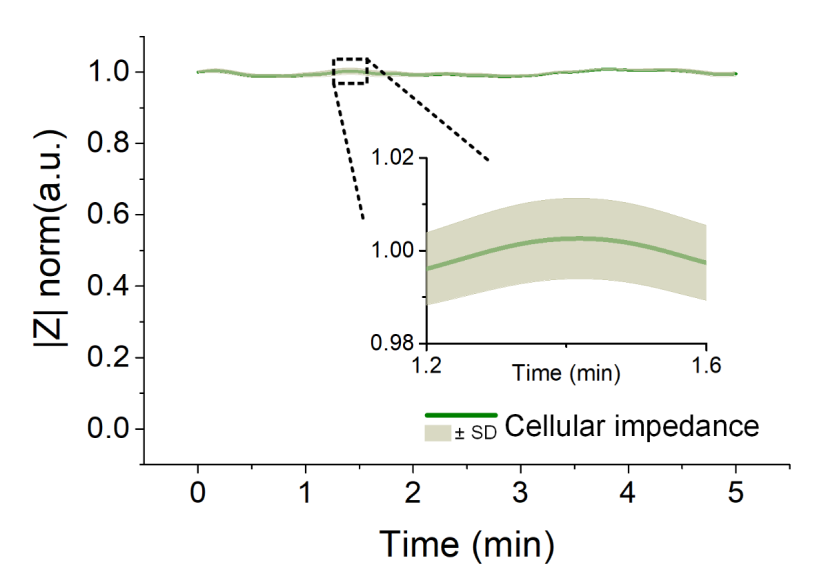


**Figure S6.** Impedance of cells without electroporation. The inset is enlargement of the partial impedance curve. The impedance of the cellswithout electroporation remainedsteady.

**Note 7. Fluorescence images of HeLa cell electroporation**


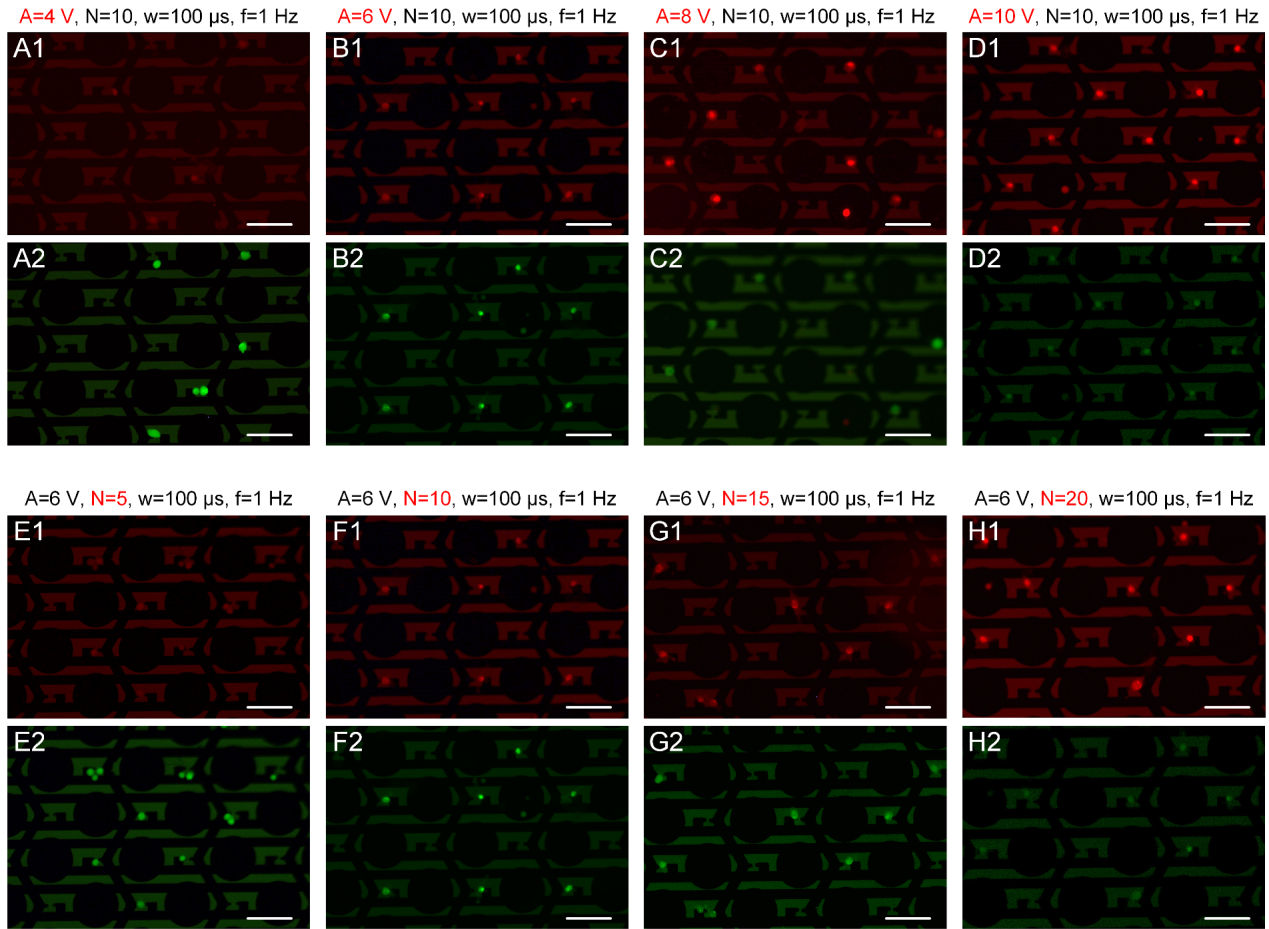


**Figure S7.** Fluorescence images of HeLa cell electroporation. The red fluorescence images were the results of PI staining. The green fluorescence images were the results of Calcein-AM staining. Scale bars were 100 μm.

**Note 8. Enlarged images of the EGFP transfection results**


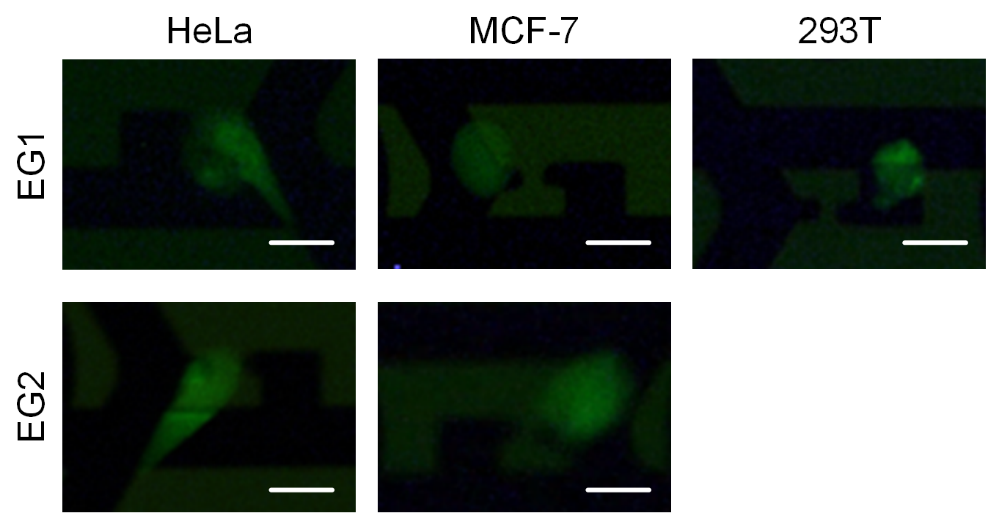


**Figure S8.** Enlarged images of the EGFP transfection results. Scale bars were 20 μm.

**Note 9. The gene map of the EGFP plasmid**


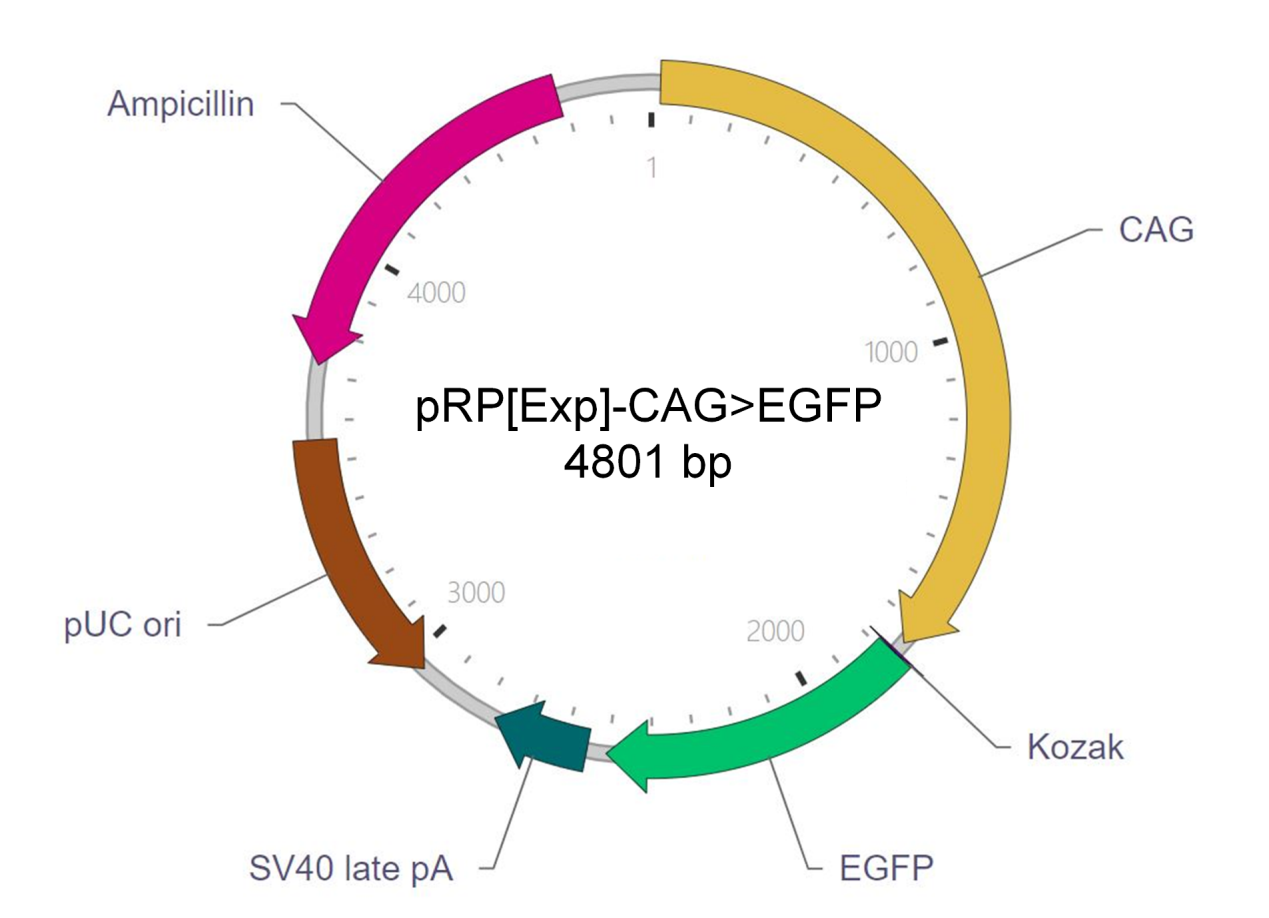


**Figure S9.** The gene map of the EGFP plasmid.

Supplementary Video

The video of single HeLacells positioning process. The signal phase difference is 180º, the peak-to-peak voltage is 2.8 Vpp, the frequency is 100 kHz. Cells have been stained with Calcien-AM to facilitate observation.
